# Prospective Survey of Postoperative Pain in Japan: A Multicenter, Observational Study

**DOI:** 10.3390/jcm14041130

**Published:** 2025-02-10

**Authors:** Masaki Kaibori, Kengo Yoshii, Tung Thanh Lai, Hideyuki Matsushima, Wataru Tatsuishi, Ryo Inada, Yasuhiro Matsugu, Koji Komeda, Mitsuhiro Asakuma, Keitaro Tanaka, Hiroshi Sato, Takeshi Yamada, Toshimitsu Miyasaka, Yutaka Hasegawa, Ryota Matsui, Kazuhiro Takehara, Saiho Ko, Ichiro Yamato, Naohiro Washizawa, Hideki Taniguchi, Yutaka Kimura, Nobuya Ishibashi, Yoshito Akagi, Naoko Hiki, Tadashi Higuchi, Tatsushi Shingai, Takashi Kamei, Hiroshi Okamoto, Yuichi Nagakawa, Chie Takishita, Takayuki Kohri, Kosuke Matsui, Yoshihiro Nabeya, Kazuhiko Fukatsu, Go Miyata

**Affiliations:** 1 Department of Hepatobiliary Surgery, Kansai Medical University, Osaka 573-1010, Japan; laithanhtung@hmu.edu.vn (T.T.L.); h.matsushima0921@gmail.com (H.M.); matsuik@hirakata.kmu.ac.jp (K.M.); 2 Department of Mathematics and Statistics in Medical Sciences, Kyoto Prefectural University of Medicine, Kyoto 602-8566, Japan; yoshii-k@koto.kpu-m.ac.jp; 3 Department of Surgery, Hanoi Medical University, Hanoi 100000, Vietnam; 4 Department of Cardiovascular Surgery, Gunma University Hospital, Gunma 371-8511, Japan; wataru0812_drt@yahoo.co.jp; 5 Department of Gastroenterological Surgery, Kochi Health Sciences Center, Kochi 781-8555, Japan; ryo_inada@hotmail.com; 6 Department of Gastroenterological Surgery, Hiroshima Prefectural Hospital, Hiroshima 734-8530, Japan; yasu.matsugu@gmail.com; 7 Department of General and Gastroenterological Surgery, Osaka Medical and Pharmaceutical University Hospital, Osaka 569-8686, Japan; komeda0502@gmail.com (K.K.); mitsuhiro.asakuma@ompu.ac.jp (M.A.); keitaro.tanaka@ompu.ac.jp (K.T.); 8 Department of Gastrointestinal Surgery, Saitama Medical University International Medical Center, Saitama 350-1298, Japan; hs8401@5931.saitama-med.ac.jp; 9 Department of Gastrointestinal and Hepato-Biliary-Pancreatic Surgery, Nippon Medical School, Tokyo 113-8603, Japan; y-tak@nms.ac.jp (T.Y.); miyasaka@nms.ac.jp (T.M.); 10 Division of Cardiovascular Surgery, Gunma Prefectural Cardiovascular Center, Gunma 371-0004, Japan; hasegawa.yu@cvc.pref.gunma.jp; 11 Department of Digestive and General Surgery, Juntendo University Urayasu Hospital, Chiba 279-0021, Japan; supreme0818@gmail.com (R.M.); ktakeha@juntendo-urayasu.jp (K.T.); 12 Department of Surgery, Nara Prefecture General Medical Center, Nara 630-8581, Japan; ko@nara-hp.jp (S.K.); yamato_16@me.com (I.Y.); 13 Nutritional Therapy Center, Toho University Omori Medical Center, Tokyo143-8541, Japan; washi@med.toho-u.ac.jp; 14 Patient Support Center, Saiseikai Yokohamashi Tobu Hospital, Kanagawa 230-8765, Japan; taniguchihideki@outlook.jp; 15 Department of Surgery, Kindai University Nara Hospital, Nara 630-0293, Japan; you-kimura@med.kindai.ac.jp; 16 Department of Surgery, Kurume University School of Medicine, Fukuoka 830-0011, Japan; hoopybaron0814@gmail.com (N.I.); yoshisg@med.kurume-u.ac.jp (Y.A.); 17 Department of Upper Gastrointestinal Surgery, Kitasato University School of Medicine, Kanagawa 252-0373, Japan; nhiki@med.kitasato-u.ac.jp (N.H.); tadashihiguchi1012@yahoo.co.jp (T.H.); 18 Department of Surgery, Saiseikai Senri Hospital, Osaka 565-0862, Japan; shingaiopen@gmail.com; 19 Department of Surgery, Tohoku University Graduate School of Medicine, Sendai 980-8575, Japan; tkamei@med.tohoku.ac.jp (T.K.); hi_ok_0531@yahoo.co.jp (H.O.); 20 Department of Gastrointestinal and Pediatric Surgery, Tokyo Medical University, Tokyo 160-8402, Japan; naga@tokyo-med.ac.jp (Y.N.); c.takishita@gmail.com (C.T.); 21 Department of Surgery, Tone Chuo Hospital, Numata 378-0012, Japan; takakohri@gmail.com; 22 Division of Esophago-Gastrointestinal Surgery, Chiba Cancer Center, Chiba 260-8717, Japan; ynabeya@chiba-cc.jp; 23 Surgical Center, The University of Tokyo Hospital, Tokyo 113-8655, Japan; fukatsu-1su@h.u-tokyo.ac.jp; 24 Department of Digestive Surgery, Iwate Prefectural Central Hospital, Iwate 020-0066, Japan; miyata5@chuo-hp.jp

**Keywords:** postoperative pain, analgesia, seven surgical procedures, Prince Henry Pain Scale, QoR-15, postoperative hospital stay

## Abstract

**Background/Objectives**: Postoperative analgesia is important for reducing biologically invasive reactions to surgery. In Japan, postoperative analgesia, including indices of analgesia, has not been adequately addressed. This study aimed to determine the relationship between postoperative pain and postoperative course and the importance of analgesia for early recovery. **Methods**: Patients who underwent any of seven surgical procedures in gastrointestinal, thoracic, and cardiac surgery were enrolled. The primary endpoint was a median Prince Henry Pain Scale score from postoperative days 1 to 3. Secondary endpoints were the quality of recovery on postoperative day 7 (Quality of Recovery-15 [QoR-15]) and the length of postoperative hospital stay. **Results**: Median postoperative pain levels among surgeries were 3 on day 1, 2 on days 2 and 3, 1 on day 7, and 1 at discharge. In both univariate and multivariate analyses, the use of postoperative epidural analgesia and intravenous patient-controlled analgesia (IV-PCA) were significant predictors of early postoperative pain. Only early postoperative pain was a significant predictor of QoR-15 score. Regular use of acetaminophen, early postoperative pain, no appetite, and postoperative complications were significant in affecting the length of postoperative hospital stay. In the comparison of early postoperative pain according to whether epidural analgesia and IV-PCA were used, the group that used both methods had the least pain. **Conclusions**: In Japan, early postoperative pain persists after major surgical procedures and affects postoperative quality of recovery and length of hospital stay. The use of epidural analgesia, IV-PCA, or both appeared to be effective in overcoming early postoperative pain, thereby enhancing early postoperative recovery.

## 1. Introduction

The Enhanced Recovery After Surgery (ERAS^®^) protocol developed in Northern Europe [1] will soon be implemented globally as a standardized approach for perioperative management [2,3,4]. However, simply adding this protocol to daily clinical practice will not guarantee success. Surgical staff must give careful consideration to post-surgical metabolic fluctuations in patients. Above all, perioperative management must be acceptable to the patient. In response to challenges in implementing ERAS, the Japanese Society for Surgical Metabolism and Nutrition (JSSMN) developed the Essential Strategy for Early Normalization after Surgery with Patient’s Excellent Satisfaction (ESSENSE) program in 2012 [5]. ESSENSE and ERAS have similar goals, but ESSENSE offers a more contextually appropriate approach for Japan, based on four key concepts: (1) the modulation of biological responses to surgical insults; (2) the early restoration of physical activity; (3) the early recovery of normal nutritional intake; and (4) the mitigation of perioperative anxiety and encouragement of enhancement of the motivation to recover. The ESSENSE program has been introduced across several types of gastroenterological surgery in Japan [5], and despite initial challenges, has been adopted in many institutions [6,7]. When used in specific gastroenterological surgeries, it aims to eliminate unnecessary and potentially harmful conventional perioperative management practices, reduce postoperative pain, and promote rapid recovery of daily activities and return to work. Achieving these goals requires pain management to serve as an essential link connecting the objectives of the ESSENSE protocol. Postoperative analgesia is an important measure to reduce biologically invasive reactions to surgery, thereby supporting the other elements of the protocol and enhancing early recovery [8]. We hypothesized that a well-structured postoperative pain management strategy, particularly for early postoperative pain, will contribute significantly to both the quality and speed of patient recovery. However, in Japan, postoperative analgesia, including indices of analgesia, has not yet been adequately addressed. As part of the ESSENSE project aimed at early postoperative recovery, the JSSMN conducted a multi-institutional prospective observational study to evaluate postoperative analgesia methods and changes in pain after various surgical procedures. In this clinical study, we aimed to examine the relationship between the degree of postoperative pain and the postoperative course and to clarify the significance of analgesia for early postoperative recovery.

## 2. Materials and Methods

### 2.1. Population

This was a multi-institutional, prospective, observational study conducted from January 2021 to December 2023. The subjects were patients who underwent gastrointestinal, thoracic, and cardiac surgery. The following surgical procedures were included in gastrointestinal surgery: (1) Esophagectomy, (2) Total gastrectomy and gastrectomy, (3) Colectomy and rectal resection, (4) Hepatectomy, (5) Pancreaticoduodenectomy. Pulmonary resection was included in thoracic surgery. The following surgical procedures were included in cardiac surgery: (1) Cardiothoracic large vessel surgery (valvular disease, ischemic heart disease, thoracic aortic surgery, congenital, etc.), (2) Abdominal large vessel surgery (aortic aneurysm, arteriosclerosis obliterans, etc.).

The inclusion criteria were as follows: (1) Patients of any gender, (2) Patients who provided their own voluntary consent after receiving and understanding a full explanation of the study, and (3) Patients treated at facilities that are members of the council of the JSSMN that perform more than 30 surgeries of the same category per year within the defined categories.

The exclusion criteria were as follows: (1) Patients undergoing surgical procedures not listed in the inclusion criteria, (2) Patients treated at facilities that did not meet the qualification standards (performing fewer than 30 surgeries of the same category per year), and (3) Patients who did not provide voluntary informed consent to participate.

The number of participating cases was about 500 in total from facilities that were members of the council of JSSMN and agreed to participate.

### 2.2. Outcomes

The primary endpoint was the median Prince Henry Pain Scale score [9,10,11] from postoperative days 1 to 3. The Prince Henry Pain Scale was used to assess pain and was divided into five stages: 0: No pain when coughing, 1: Pain when coughing, but not when taking a deep breath, 2: Pain when taking a deep breath, but no pain at rest, 3: Pain at rest, but no painkillers needed, 4: Pain at rest, and painkillers needed. The Prince Henry Pain Scale is an activity-based pain assessment scale that is well-suited for evaluating postoperative patients and adjusting analgesic dosing, especially for those who need to resume activities early as part of the ERAS protocol. Pain assessments were conducted on the 1st, 2nd, 3rd, and 7th days after surgery and at the time of discharge. Pain was defined as the worst pain, including at the drain insertion site, and the worst pain on any given day was recorded through patient interview. Physicians at each facility explained the differences between these stages before and after surgery so that they were understood by the patients.

Secondary endpoints were quality of postoperative recovery on the 7th day after surgery (Quality of Recovery-15 [QoR-15]) [12,13] and length of postoperative hospital stay (days).

The survey items were as follows: (1) patient information, (2) method of general anesthesia, (3) postoperative analgesic measures, (4) other evaluation items that directly or indirectly impact postoperative recovery or serve as indicators of the recovery process, including time to start eating, degree of appetite and ambulation, presence or absence of postoperative complications, presence or absence of postoperative nausea/vomiting within 2 days after surgery, presence or absence of postoperative intestinal paralysis from the 3rd day after surgery onwards, length of postoperative hospital stay, time of first flatus, time of first defecation, and perioperative measurements of total lymphocyte count, neutrophil count, serum albumin, and serum C-reactive protein (CRP) levels. Postoperative complications were defined as those of Clavien–Dindo classification grade III or higher [14]. The study protocol was approved by the institutional ethics committee of Kansai Medical University (reference number: KMU 2021102). Ethics applications were also submitted and approved at each participating institution. Informed consent for this study was obtained from each participating institution.

### 2.3. Statistical Analysis

Quantitative variables were expressed as medians (interquartile ranges), and qualitative variables were expressed as frequencies and percentages. Correlations between the Prince Henry Pain Scale score, QoR-15 score, length of postoperative hospital stay, and clinical factors were evaluated using Spearman’s rank correlation coefficient. Comparisons of qualitative clinical factors related to the Prince Henry Pain Scale score, QoR-15 score, and length of postoperative hospital stay were performed using the Wilcoxon rank sum test. In addition, the Prince Henry Pain Scale score, QoR-15 score, and length of postoperative hospital stay were dichotomized at the median, and associated risk factors were examined using univariate and multivariate logistic regression analyses. Group comparisons of clinical characteristics selected from the multivariate analysis were performed using the Wilcoxon rank sum test, while the Steel–Dwass test was used for multiple comparisons. *p* values less than 0.05 were considered significant. All data analyses were conducted using the R statistical package (version 4.3.3, R Foundation for Statistical Computing, Vienna, Austria).

## 3. Results

### 3.1. Patient Background for Seven Organ Surgeries

A total of 593 patients underwent surgery on one of seven organs, and 25 facilities participated in this study (Table 1). The median age was 72, and there were more males than females in each organ surgery group. There were no surgeries for malignant tumors in the cardiovascular surgery group. Of the 593 total surgeries, 366 were laparoscopic, thoracoscopic, or robot-assisted surgeries.

### 3.2. Changes in Postoperative Pain Following Seven Organ Surgeries

**Figure 1:**
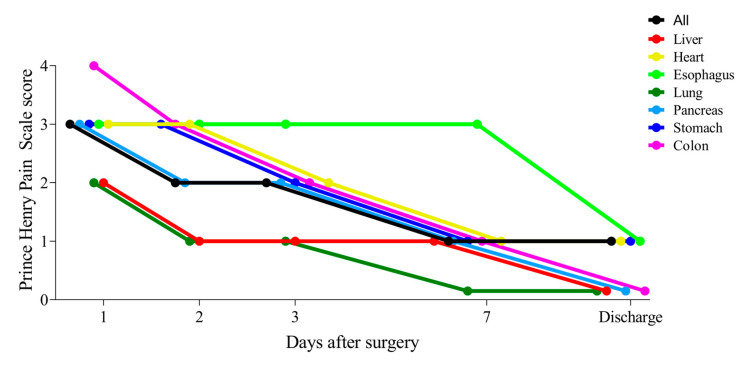
Changes in postoperative pain following seven organ surgeries. Postoperative pain level was assessed using the Prince Henry Pain Scale score on postoperative days 1, 2, 3, 7, and at discharge, with five levels: 0: No pain when coughing, 1: Pain when coughing, but not when taking a deep breath, 2: Pain when taking a deep breath, but no pain at rest, 3: Pain at rest, but no painkillers needed, 4: Pain at rest, and painkillers needed.

The median (interquartile range) postoperative pain level according to the Prince Henry Pain Scale score in the seven organ surgeries was as follows: 3 (2–4) on the first day, 2 (1–3) on the second day; 2 (1–3) on the third day; 1 (1–1) on the seventh day; and 1 (0–1) at the time of discharge (Figure 1). The postoperative hospital stay in seven organ surgeries was 11 (8–15) days. The pain intensity was highest during the first three postoperative days and gradually decreased by the seventh day, with nearly minimal levels at discharge across all surgical groups. Pain after colon surgery was severe on the first postoperative day, but from the second postoperative day onwards, it showed a similar progression to heart, esophagus, pancreas, and stomach surgeries. In contrast, pain after lung and liver surgeries was relatively low up to the third postoperative day.

### 3.3. Correlations Between Clinical Factors and the Three Endpoints

Factors strongly correlated with the median pain level on postoperative days 1–3 were food intake ratio throughout the entire postoperative course, QoR-15 score on the 7th day after surgery, postoperative hospital stay, and perioperative neutrophil count (Table 2). The following eight factors were strongly correlated with the QoR-15 score on the seventh postoperative day: amount of blood loss during surgery, operation time, length of wound, pain on postoperative days 1–3, food intake ratio and five-level appetite throughout the entire postoperative course, postoperative hospital stay, and perioperative neutrophil count. The following 13 factors were strongly correlated with postoperative hospital stay: blood loss during surgery, operation time, length of wound, urinary volume during surgery, pain on postoperative days 1–3, onset of eating, food intake ratio, five-level appetite and 10 levels of bed exit throughout the entire postoperative course, QoR-15 score on postoperative day 7, and perioperative neutrophil count, and serum albumin and CRP levels.

### 3.4. Comparison of Clinical Factors on the Three Endpoints

When comparing clinical factors on the Prince Henry Pain Scale score, QoR-15 score, and postoperative hospital stay, significant differences were found on all three endpoints: American Society of Anesthesiologists Physical Status (ASA-PS) classification (I, II, and III), the presence or absence of intestinal paralysis, and the presence or absence of postoperative epidural analgesia (Table 3).

### 3.5. Univariate and Multivariate Logistic Regression Analyses of Risk Factors for the Three Endpoints

Results of the logistic regression analysis examining risk factors for Prince Henry Pain Scale score are presented in Table 4A. The use of postoperative epidural analgesia and IV-PCA were identified as significant predictors in both the univariate and multivariate analyses. Regarding the investigation of risk factors for the QoR-15 score (Table 4B), only a Prince Henry Pain Scale score ≥ 2.33 was identified as a significant predictor in both the univariate and multivariate analyses. The following four factors were found to be significant in affecting the length of postoperative hospital stay: regular use of acetaminophen, Prince Henry Pain Scale score ≥ 2.33, Five levels of appetite ≥ 2.75, and the occurrence of postoperative complications (Table 4C).

### 3.6. Effects of Risk Factors Selected from Multivariate Analyses on Each Endpoint

**Figure 2:**
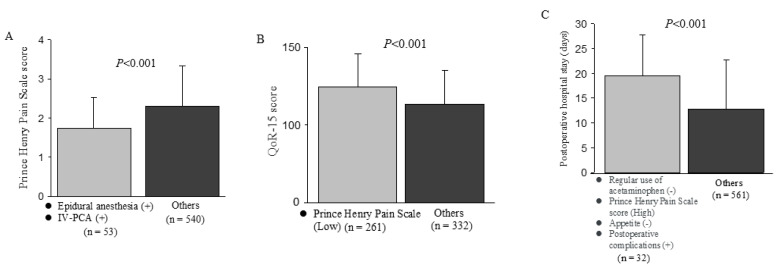
Effects of risk factors selected from multivariate analyses on each endpoint: (**A**) Prince-Henry Pain Scale score, (**B**) QOR-15 score, (**C**) Postoperative hospital stay. *p* values were calculated using the Wilcoxon rank sum test.

Patients who received both epidural analgesia and IV-PCA after surgery had significantly less early postoperative pain than those who did not (Figure 2A). The group with low early postoperative pain showed higher QoR-15 scores on the seventh day after surgery compared with the other groups (Figure 2B). The 32 patients who did not take acetaminophen regularly had early postoperative pain and no appetite after surgery, and developed postoperative complications had a significantly longer hospital stay than the other 561 patients (Figure 2C).

**Figure 3:**
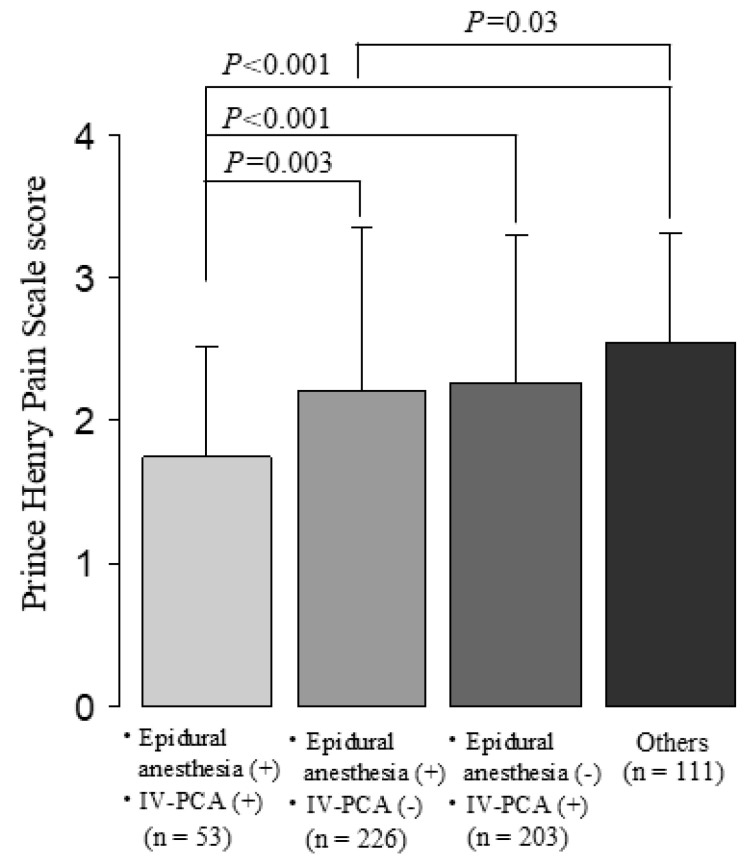
Degree of early postoperative pain with or without postoperative epidural anesthesia and/or IV-PCA. The table below shows the number of patients who used epidural analgesia and/or IV-PCA for each organ surgery. IV-PCA; intravenous patient-controlled analgesia. *p* values were calculated using the Steel–Dwass test for multiple comparisons.

We investigated the degree of early postoperative pain according to whether postoperative epidural analgesia and/or IV-PCA were used. The group using both methods had the least amount of pain in the early postoperative period, while the group not using either method had the most severe pain (Figure 3). These 53 patients who were provided with both epidural analgesia and IV-PCA had the most reduced early postoperative pain. Both methods are not generally used at the same time, and it is presumed that IV-PCA was initiated as a support when the effect of epidural anesthesia was insufficient. In the group not using either method, 64 of 111 patients used acetaminophen alone, 39 used acetaminophens in combination with NSAIDs, and 8 used NSAIDs alone.

## 4. Discussion

The four core concepts of ESSENSE, the Japanese version of ERAS, are the following: (1) the modulation of biological responses to surgical insults; (2) the early restoration of physical activity; (3) the early recovery of normal nutritional intake; and (4) the mitigation of perioperative anxiety and encouragement of enhancement of the motivation to recover [5]. The underlying element “modulation of biological responses to surgical insults” informs the other elements, all of which are directed toward quick postoperative recovery. The most important and central item among these four concepts is the suppression and prevention of postoperative pain. In other words, if the patient’s pain is suppressed, it is assumed that they will be able to walk, have an appetite, eat better, have bowel movements, and feel more positive [8]. In our study, we investigated perioperative outcomes focusing on postoperative pain at representative facilities for various surgeries in Japan. This highlighted the fact that postoperative pain in various surgeries still occurs frequently in the early postoperative period (Figure 1), a situation that has not been overcome with our perioperative management. It was found that the use of epidural analgesia, IV-PCA, or both, was effective in suppressing this early postoperative pain (Figure 3). On the other hand, early postoperative pain significantly affected patients’ satisfaction with their hospital stay in the first week after surgery and their length of postoperative hospital stay (Table 4). QOR-15, an indicator of postoperative patient satisfaction, is a sensitive reflection of the patient’s postoperative condition and correlates with the occurrence of postoperative complications, making it an important screening tool for observing the “quality” of the postoperative course [13]. In our study, Table 2 shows the correlations between the Prince Henry Pain Scale score, QoR-15 score, postoperative hospital stays, and clinical factors. Among these, the factor that most influenced QOR-15 was early postoperative pain, highlighting that effective pain management strategies can significantly improve recovery quality. This conclusion is further supported by the finding that a Prince Henry Pain Scale score ≥ 2.33 was identified as a significant predictor in both the univariate and multivariate analyses (Table 4B). Correlations between QoR-15 scores and surgical variables, including operative blood loss, operation time, and length of wound, indicate that use of perioperative management strategies, such as minimizing blood loss and optimizing surgical techniques, could enhance recovery quality. In addition, shortening the postoperative hospital stay by achieving early recovery is an important issue in hospital management in terms of increasing the efficiency of bed utilization. Japan has a system where all citizens can receive medical insurance, which is different from the insurance systems in the U.S. and Europe. This study revealed that to shorten postoperative hospital stay, it is important to reduce early postoperative pain, that regular administration of acetaminophen and/or NSAIDs after surgery is recommended [15,16,17], that these efforts increase appetite, and early mobilization, and, naturally, that the occurrence of postoperative complications is decreased.

The results of our study suggest that because early postoperative pain is clearly present, it will be important to provide more thorough postoperative pain management in the near future to ensure early postoperative recovery. Pain and anxiety are two of the most common factors influencing recovery from surgery [18]. When inappropriately treated, pain can cause immunosuppression, tachycardia, increased oxygen consumption, and increased catecholamine production [19]. To hasten discharge and recovery, postoperative treatments should minimize physiological and psychological stress [20]. Postoperative analgesia has been reported to be a key contributor to the postoperative management of patients who have undergone gastrointestinal surgery in ERAS programs [21]. Epidural analgesia and IV-PCA are key strategies for perioperative management of patients who undergo abdominal surgery, especially surgeries involving an upper abdominal incision [22,23]. Epidural analgesia has been recognized for many years to be superior to intravenous opioid analgesia, as it provides better pain relief at movement and early intestinal movement, and it is associated with a lower incidence of postoperative pulmonary complications [22,23]. However, the results of recent clinical studies using multimodal analgesia consisting of regional anesthesia, non-opioid analgesics, and opioids have called this superiority into question [24,25,26]. Epidural analgesia will be replaced by multimodal analgesia using peripheral regional analgesia, non-opioids, and rescue opioids for laparoscopic surgery and lower laparotomy. However, its clinical utility will not wane for pain relief after major upper abdominal laparotomy. On the other hand, IV-PCA has the advantage that the administration route is easy to establish, and it can be used in cases where epidural analgesia is not applicable. However, compared to epidural analgesia, it has disadvantages such as inferior analgesic effect during body movement, high incidence of respiratory complications, and slow recovery of gastrointestinal motility [27,28,29,30,31]. In addition, if analgesia is achieved only with IV-PCA with opioids, the side effects of the opioids may hinder postoperative recovery [30,31]. Therefore, when using IV-PCA, it is necessary to devise a method to enhance the analgesic effect while avoiding the adverse effects of opioids by combining it with other analgesic methods, multimodal analgesia. Figure 2A and Figure 3 show that the combination of epidural analgesia and IV-PCA as multimodal analgesia provided pain relief superior to that obtained with either method alone. This combination was not initially implemented; instead, IV-PCA was added in cases where epidural analgesia alone was insufficient. This addition offers the advantage of rapid onset and broader pain coverage, which may improve efficacy. Although this combination improved pain relief, careful consideration must be given to the potential side effects associated with increased opioid doses. A recent clinical trial showed that combining short-term thoracic epidural analgesia with IV-PCA on the first two postoperative days and applying IV-PCA alone on the two subsequent days resulted in superior pain relief compared to thoracic epidural analgesia alone, with no significant increase in opioid-related side effects [32].

The present study has some limitations. (1) Limited case numbers per surgical procedure: the small sample size for each procedure suggests that additional data per technique would improve the clinical relevance of findings. Analyzing postoperative pain for each surgical method independently might provide greater insight. An increased number of cases for each procedure, adoption of a standardized pain management protocol, and pain assessments for individual procedures are warranted in future studies. (2) It is necessary to clarify the rationale for selecting cases in which epidural analgesia or IV-PCA was administered, as well as the impact of the analgesic drug doses of each method on the level of pain. In the present study, it was presumed that anesthesiologists at each institution followed their own institution’s practice. (3) The Prince Henry Pain Scale was used in this study. It is suitable for evaluating postoperative pain related to physical activity; however, it is less commonly used than the Numeric Rating Scale. This limits its comparability with other studies. In addition, this scale offers only five levels and does not consider the psychological or emotional aspects of pain, making it less sensitive for comprehensive pain assessment, especially in studies that require higher-resolution data. To address this, other pain assessments should have been performed simultaneously, as this scale may not be sufficient to capture comprehensive pain results.

## 5. Conclusions

It was clear that early postoperative pain persists after major surgical procedures in Japan. Early postoperative pain affected the postoperative quality of recovery and length of hospital stay. The use of epidural analgesia, IV-PCA, or both appeared to be effective in overcoming early postoperative pain, thereby enhancing early postoperative recovery.

## Figures and Tables

**Table 1:** Patient background for seven organ surgeries.

Variable	Liver		Cardiovascular		Esophagus		Lung		Pancreas		Stomach		Colorectal	
Number of patients	105		110		32		30		62		66		188	
Number of participating hospitals	3		2		2		2		4		5		7	
Age	72 (65–77)		72 (65–76)		71 (63–74)		71 (69–76)		73 (66–77)		74 (68–79)		71 (60–79)	
Gender (male/female)	72 (69%)/33 (31%)		84 (76%)/26 (24%)		25 (78%)/7 (22%)		20 (67%)/10 (33%)		35 (56%)/27 (44%)		44 (67%)/22 (33%)		103 (55%)/85 (45%)	
Diagnosis	Hepatocellular carcinoma	47	Aortic disease	18	Esophageal cancer	32	Lung cancer	25	Pancreatic cancer	42	Gastric cancer	65	Right side colon cancer	68
	Metastatic tumor	44	Valvular disease	35			Metastatic tumor	3	Cholangiocarcinoma	6	Others	1	Left side colon cancer	59
Diagnosis	Cholangiocarcinoma	9	Ischemic heart disease	25			Others	2	Duodenal cancer	7			Rectal cancer	54
	Others	5	AAA	32					NET or benign disease	7			Inflammatory bowel disease	3
													Others	4
Stage (UICC)														
I	12 (11%)		–		5 (16%)		19 (63%)		21 (34%)		42 (64%)		47 (25%)	
II	32 (30%)		–		11 (34%)		7 (23%)		31 (50%)		11 (17%)		61 (32%)	
III	9 (9%)		–		13 (41%)		0 (0%)		3 (5%)		7 (11%)		61 (32%)	
IV	47 (45%)		–		3 (9%)		2 (7%)		0 (0%)		5 (7%)		12 (7%)	
No malignancy	5 (4%)		110 (100%)		0 (0%)		2 (7%)		7 (11%)		1 (1%)		7 (4%)	
Surgical Procedures	Partial hepatectomy	14	Thoracic aortic surgery	17	Thoracoscopic esophagectomy	30	Thoracoscopic pulmonary resection	12	Pancreatoduodenectomy	40	Distal gastrectomy	8	Laparoscopic colectomy	120
	Segmentectomy	19	Valve replacement	36	Robotic-assisted	2	Robotic-assisted	18	Distal pancreatectomy	4	Total gastrectomy	3	Robotic-assisted colectomy	9
	Sectionectomy	9	CABG	25					Laparoscopic DP	3	Laparoscopic DG	33	Laparoscopic proctectomy	38
	Bisectionectomy	12	Y-grafting	32					Robotic-assisted PD	10	Laparoscopic TG	3	Robotic-assisted proctectomy	13
	Laparoscopic partial hepatectomy	27							Robotic-assisted DP	4	Robotic-assisted gastrectomy	18	Abdominoperineal resection of rectum	3
	Laparoscopic segmentectomy	17							Total pancreatectomy	1	Laparoscopic PG	1	Open colectomy	4
	Laparoscopic sectionectomy	3											Laparoscopic small intestine resection	1
	Laparoscopic bisectionectomy	4												

Data are shown as median (interquartile range) or n (%). NET, neuroendocrine tumor; AAA, abdominal aortic aneurysm; DP, distal pancreatectomy; PD, pancreatoduodenectomy; CABG, Coronary artery bypass grafting; DG, distal gastrectomy; TG, total gastrectomy; PG, proximal gastrectomy.

**Table 2:** Correlations between Prince Henry Pain Scale score, QoR-15 score, postoperative hospital stay, and clinical factors ^a^.

Variable	Prince Henry Pain Scale Score		QoR-15 Score		Postoperative Hospital Stay (Days)	
	Correlation Coefficient (ρ)	*p*	Correlation Coefficient (ρ)	*p*	Correlation Coefficient (ρ)	*p*
Age (years)	−0.09	0.030	−0.04	0.335	0.12	0.005
Operative blood loss (mL)	−0.04	0.375	−0.18	<0.001	0.30	<0.001
Operative time (min)	0.03	0.513	−0.19	<0.001	0.41	<0.001
Length of wound (cm)	−0.05	0.228	−0.25	<0.001	0.36	<0.001
Urine volume (mL)	−0.06	0.129	−0.08	0.075	0.24	<0.001
Prince Henry Pain Scale	–	–	−0.31	<0.001	0.20	<0.001
First gas discharge (days after surgery)	0.05	0.223	−0.03	0.553	0.11	0.011
First bowel movement (days after surgery)	−0.01	0.752	−0.01	0.791	0.11	0.011
Start of eating (days after surgery)	0.12	0.004	−0.03	0.481	0.31	<0.001
Food intake ratio (%)	−0.16	<0.001	0.25	<0.001	−0.26	<0.001
Five levels of appetite	−0.11	0.022	0.26	<0.001	−0.37	<0.001
10 levels of bed exit	0.01	0.802	0.11	0.011	−0.33	<0.001
QOR-15 score on the 7th day after surgery	−0.31	<0.001	–	–	−0.39	<0.001
Postoperative hospital stays (days)	0.20	<0.001	−0.39	<0.001	–	–
Perioperative total lymphocyte count (/μL)	−0.19	0.001	0.19	0.001	−0.15	0.006
Perioperative neutrophil count (/μL)	0.25	<0.001	−0.30	<0.001	0.27	<0.001
Perioperative serum albumin level (g/dL)	0.05	0.373	0.16	0.004	−0.23	<0.001
Perioperative serum CRP level (mg/dL)	0.06	0.262	−0.16	0.003	0.27	<0.001

QoR: Quality of Recovery; CRP: C-reactive protein. Note: *p* values were calculated using the Wilcoxon rank sum test. ^a^ Correlations were evaluated using Spearman’s rank correlation coefficient.

**Table 3:** Comparison of effects of clinical factors on the Prince Henry Pain Scale score, QoR-15 score, and postoperative hospital stay ^a^.

Variable	Prince Henry Pain Scale Score			QoR-15 Score			Postoperative Hospital Stay (Days)		
	Median	(IQR)	*p*	Median	(IQR)	*p*	Median	(IQR)	*p*
Gender			0.027			0.221			0.043
Male	2.3	(1.3, 3.0)		124	(106, 136)		11	(8, 16)	
Female	2.3	(1.7, 3.3)		120	(102, 134)		10	(8, 14)	
Preoperative treatment (chemotherapy, radiation, etc.)			0.600			0.005			<0.001
No	2.3	(1.3, 3.0)		123	(107, 136)		10	(8, 15)	
Yes	2.3	(1.3, 3.0)		115	(97, 129)		14	(11, 20)	
Laparoscopic or thoracic surgery			0.336			<0.001			<0.001
No	2.3	(1.7, 3.0)		115	(100, 127)		12	(9, 18)	
Yes	2.3	(1.3, 3.0)		128	(110, 139)		10	(7, 13)	
ASA-PS Classification			0.016			<0.001			<0.001
I, II	2.3	(1.3, 3.0)		126	(107, 137)		10	(8, 14)	
III	2.7	(2.0, 3.3)		112	(99, 125)		14	(10, 18)	
Postoperative complication			0.118			<0.001			<0.001
No	2.3	(1.3, 3.0)		125	(109, 137)		9	(7, 13)	
Yes	2.3	(1.7, 3.0)		109	(91, 127)		18	(13, 26)	
PONV			0.141			0.228			0.871
No	2.3	(1.3, 3.0)		123	(106, 136)		11	(8, 15)	
Yes	2.3	(1.7, 3.3)		120	(100, 131)		11	(8, 16)	
Intestinal paralysis			0.017			0.001			<0.001
No	2.3	(1.3, 3.0)		123	(106, 135)		10	(8, 15)	
Yes	2.7	(2.3, 3.3)		101	(79, 118)		18	(14, 23)	
Postoperative epidural analgesia			0.003			0.004			0.005
No	2.3	(1.7, 3.0)		119	(105, 131)		10	(8, 15)	
Yes	2.0	(1.3, 3.0)		126	(106, 139)		11	(8, 16)	
IV-PCA			0.031			0.051			0.620
No	2.3	(1.7, 3.0)		120	(105, 133)		11	(8, 15)	
Yes	2.0	(1.3, 3.0)		126	(106, 137)		10	(8, 16)	
Regular use of acetaminophen			0.046			0.223			<0.001
No	2.7	(1.7, 3.3)		126	(108, 136)		13	(9, 18)	
Yes	2.3	(1.3, 3.0)		120	(105, 135)		10	(8, 14)	

QoR: Quality of Recovery; IQR: interquartile range; ASA-PS; American Society of Anesthesiologists-Physical Status; PONV: postoperative nausea and vomiting; IV-PCA; intravenous patient-controlled analgesia. ^a^ Comparisons were performed using the Wilcoxon rank sum test.

**Table 4:** Univariate and multivariate logistic regression analyses of risk factors for Prince Henry Pain Scale score, QoR-15 score, and postoperative hospital stay in various organ surgeries.

**(A) Prince Henry Pain Scale score**				
**Variable**	**Univariate Analysis**		**Multivariate Analysis**	
	**OR (95% CI)**	***p***	**OR (95% CI)**	***p***
Age ≥ 72 years (vs. <72 years)	0.97 (0.70–1.34)	0.838	1.00 (0.92–1.08)	0.957
Gender Female (vs. Male)	1.34 (0.95–1.90)	0.095	1.06 (0.98–1.16)	0.161
Preoperative treatment Yes (vs. No)	1.08 (0.66–1.77)	0.761	1.11 (0.96–1.29)	0.169
Laparoscopic or thoracic surgery Yes (vs. No)	0.79 (0.57–1.11)	0.182	1.10 (0.84–1.44)	0.496
Operative blood loss ≥ 80.0 mL (vs. <80.0 mL)	0.99 (0.71–1.37)	0.947	1.03 (0.91–1.17)	0.660
Operative time ≥ 282 min (vs. <282 min)	1.27 (0.91–1.77)	0.155	0.99 (0.90–1.09)	0.860
Length of wound ≥ 12.85 cm (vs. <12.85 cm)	1.10 (0.78–1.56)	0.585	1.12 (0.85–1.47)	0.439
ASA-PS III (vs. I, II)	1.88 (1.25–2.85)	0.003	1.05 (0.92–1.20)	0.498
Postoperative epidural analgesia Yes (vs. No)	0.52 (0.37–0.73)	<0.001	0.71 (0.62–0.81)	<0.001
IV-PCA Yes (vs. No)	0.70 (0.50–0.98)	0.039	0.74 (0.66–0.83)	<0.001
Regular use of acetaminophen Yes (vs. No)	0.84 (0.55–1.26)	0.399	1.01 (0.91–1.12)	0.892
**(B) QoR-15 score**				
**Variable**	**Univariate Analysis**		**Multivariate Analysis**	
	**OR (95% CI)**	***p***	**OR (95% CI)**	***p***
Laparoscopic or thoracic surgery Yes (vs. No)	2.98 (2.09–4.26)	<0.001	4.01 (0.74–21.70)	0.107
Operative blood loss ≥ 80.0 mL (vs. <80.0 mL)	0.49 (0.35–0.69)	<0.001	1.06 (0.37–3.01)	0.912
Operative time ≥ 282 min (vs. <282 min)	0.65 (0.46–0.91)	0.012	1.69 (0.80–3.59)	0.172
Length of wound ≥ 12.85 cm (vs. <12.85 cm)	0.30 (0.21–0.44)	<0.001	0.98 (0.14–6.93)	0.980
ASA-PS III (vs. I, II)	0.38 (0.25–0.58)	<0.001	1.17 (0.40–3.43)	0.781
Prince Henry Pain Scale ≥ 2.33 (vs. <2.33)	0.33 (0.23–0.47)	<0.001	0.39 (0.18–0.82)	0.013
Mean food intake ratio ≥ 32.5% (vs. <32.5%)	1.59 (1.11–2.29)	0.011	2.09 (0.92–4.74)	0.079
Five levels of appetite ≥ 2.75 (vs. <2.75)	1.87 (1.46–2.42)	<0.001	1.33 (0.86–2.07)	0.202
Postoperative complication Yes (vs. No)	0.40 (0.26–0.60)	<0.001	0.52 (0.25–1.09)	0.084
Postoperative hospital stays ≥ 11 days (vs. <11 days)	0.32 (0.22–0.45)	<0.001	0.60 (0.27–1.33)	0.209
Perioperative neutrophil count ≥ 7847.5 (vs. <7847.5 (/μL))	0.28 (0.18–0.44)	<0.001	0.57 (0.29–1.13)	0.108
**(C) Postoperative hospital stay (days)**				
**Variable**	**Univariate Analysis**		**Multivariate Analysis**	
	**OR (95% CI)**	***p***	**OR (95% CI)**	***p***
Preoperative treatment Yes (vs. No)	3.39 (2.00–6.01)	<0.001	0.85 (0.28–2.60)	0.774
Operative blood loss ≥ 80.0 mL (vs. <80.0 mL)	2.77 (1.98–3.89)	<0.001	1.45 (0.61–3.44)	0.403
Urine volume ≥ 300 mL (vs. <300 mL)	2.06 (1.48–2.88)	<0.001	1.51 (0.77–2.95)	0.234
ASA-PS III (vs. I, II)	3.28 (2.15–5.10)	<0.001	2.32 (0.54–9.92)	0.257
Regular use of acetaminophen Yes (vs. No)	0.53 (0.34–0.80)	0.003	0.07 (0.02–0.31)	<0.001
Prince Henry Pain Scale ≥ 2.33 (vs. <2.33)	1.95 (1.40–2.74)	<0.001	2.30 (1.04–5.06)	0.039
Five levels of appetite ≥ 2.75 (vs. <2.75)	0.41 (0.32–0.53)	<0.001	0.45 (0.29–0.71)	<0.001
10 levels of bed exit ≥ 8.25 (vs. <8.25)	0.33 (0.23–0.48)	<0.001	0.50 (0.25–1.02)	0.058
QoR-15 score on the 7th day after surgery ≥ 122 (vs. <122)	0.32 (0.22–0.45)	<0.001	1.11 (0.53–2.32)	0.782
Postoperative complication Yes (vs. No)	14.22 (8.19–26.59)	<0.001	9.06 (3.54–23.20)	<0.001
Perioperative neutrophil count ≥ 7847.5 (vs. <7847.5 (/μL))	2.10 (1.32–3.36)	0.002	1.00 (0.47–2.15)	0.993
Perioperative serum albumin level ≥ 3.025 (vs. <3.025)	0.46 (0.28–0.72)	<0.001	0.75 (0.37–1.51)	0.418
Perioperative serum CRP level ≥ 7.70 (vs. <7.70 (mg/dL))	2.00 (1.26–3.18)	0.003	0.89 (0.44–1.77)	0.734

Multivariate regression analysis was adjusted for type of surgery (liver, heart, esophagus, lung, pancreas, stomach, and colon). ASA-PS: American Society of Anesthesiologists-Physical Status; CI, confidence interval; OR, odds ratio; IV-PCA; intravenous patient-controlled analgesia; QoR: Quality of Recovery; CRP: C-reactive protein.

## Data Availability

Due to the nature of this research, participants in this study could not be contacted regarding whether the findings could be shared publicly; thus, supporting data are not available. The datasets generated and/or analyzed for the current study are not publicly available due to the nature of the research, as noted above. Further enquiries can be directed to the corresponding author.

## Abbreviations

The following abbreviations are used in this manuscript:

ERAS	Enhanced Recovery After Surgery
ESSENSE	Essential Strategy for Early Normalization after Surgery with Patient’s Excellent satisfaction
JSSMN	Japanese Society for Surgical Metabolism and Nutrition
QoR-15	Quality of Recovery-15
CRP	C-reactive Protein
ASA-PS	American Society of Anesthesiologists Physical Status
IV-PCA	Intravenous Patient-Controlled Analgesia
